# Hémorragie méningée et hématome parenchymateux révélant une thrombose veineuse cérébrale

**DOI:** 10.11604/pamj.2015.20.293.6581

**Published:** 2015-03-25

**Authors:** Toufik Joulali, Ali Derkaoui, Sophia Besri, Mohammed Malki, Abdelkarim Shimi, Mohammed Khatouf

**Affiliations:** 1 Service de Réanimation Polyvalente A1, CHU Hassan II, Fès, Maroc

**Keywords:** Thrombophlébite cérébrale, hémorragie méningée, hématome cérébral, épilepsie, céphalées, cerebral thrombophlebitis, subarachnoid hemorrhage, cerebral hematoma, epilepsy, headache

## Abstract

La thrombophlébite cérébrale (TVC) est une cause non négligeable des accidents vasculaires cérébraux avec une grande diversité de leur présentation Clinique source d'errance et de retard thérapeutique. Nous rapportons l'observation d'une patiente présentant une TVC révélée par une hémorragie méningée. Le diagnostic a été suspecté à l'artériographie réalisée dans le cadre du bilan étiologique et thérapeutique de l'hémorragie méningée et confirmé par la suite sur l'angio-scanner. La particularité de cette observation est la difficulté aussi bien diagnostique de la TVC et sa révélation par une hémorragie méningée, que thérapeutique concernant l'utilisation des anticoagulants.

## Introduction

La thrombophlébite cérébrale ou thrombose veineuse cérébrale est une pathologie rare. Son incidence est mal connue et estimée dans certaines séries d'autopsie à moins de 2% des accidents vasculaires cérébraux. Elle touche assez souvent le sujet jeune avec une prédominance féminine 3F/1H [1]. La présentation clinique de la thrombophlébite cérébrale est très variable allant des simples céphalées aux troubles et aux complications neurologiques les plus graves [2]. Nous rapportons un cas trompeur de thrombophlébite cérébrale révélée par une hémorragie méningée associée à un hématome intra-parenchymateux avec une évolution favorable sous anticoagulation curative.

## Patient et observation

**Figure 1:**
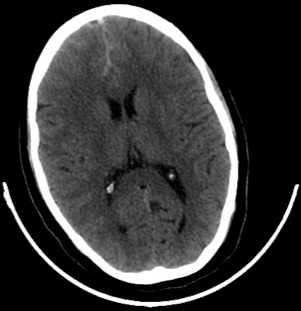
Coupe scannographique en C- objectivant une hémorragie méningée

**Figure 2:**
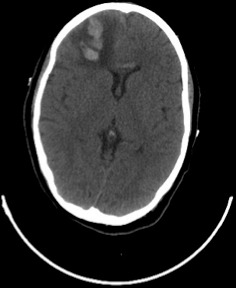
Coupe scannographique en C- montrant un hématome frontal droit avecœdème péri-lésionnel

**Figure 3:**
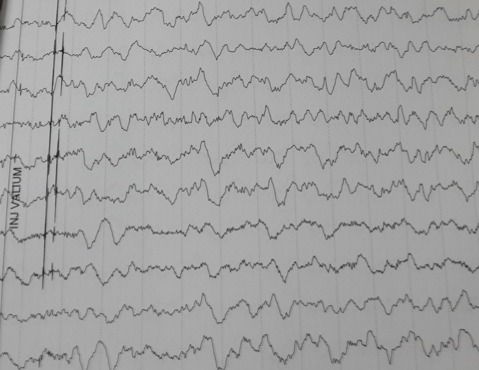
Éléctro-encéphalogramme en faveur d'un état de mal infra-clinique

**Figure 4:**
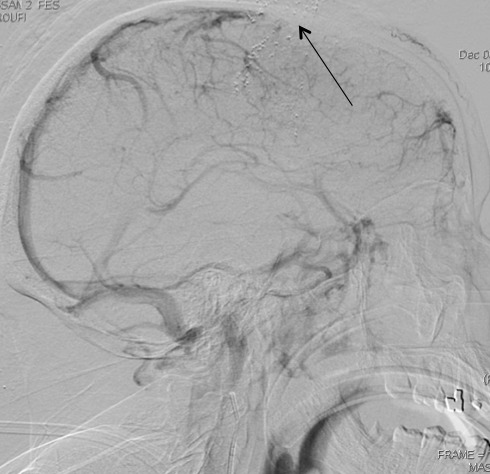
Coupe sagitale en artériographie en temps veineux objectivant un défecte de réaussement du sinus longitudinal supérieur

**Figure 5:**
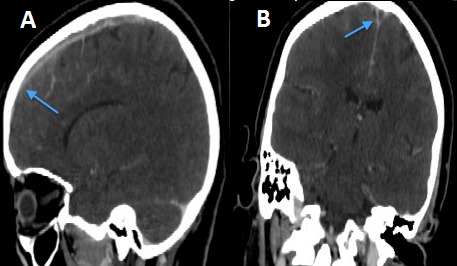
A) scanner injecté en coupe sagittale montrant le défecte du réaussement auniveau du sinus longitudinal supérieur avec ishémie du lobe frontal en regard; B) scanner injecté en coupe coronale objectivant le thrombus au niveau du sinus longitudinal supérieur

Patiente N.K, âgée de 37ans, sous contraception orale à base d'oestroprogestatifs, admise aux urgences du CHU Hassan II de Fès pour la prise en charge de troubles de conscience compliquant des céphalées sub-aigues et une photophobie évoluant depuis cinq jours. L´examen aux urgences trouve une patiente inconsciente, GCS à 11, des pupilles égales et symétriques, une hémiplégie gauche et des paramètres vitaux normaux. Le scanner initial objective une hémorragie méningée (Figure 1) pour laquelle la patiente fut mise sous nimodipine par voie injectable avec mesures de réanimation. Le bilan biologique est normal, l´électrocardiogramme et la radiographie du thorax sans anomalie. L´évolution est marquée par l´apparition de crises convulsives tonico-cloniques suivies d'un coma post-critique. Au scanner cérébral de contrôle on objective l´apparition d´un hématome frontal associé à un oedème péri-lésionnel (Figure 2) tandis que l´éléctro-encéphalogramme cérébral a montré un état de mal infra-clinique avec un rythme de fond ralenti et une souffrance cérébrale diffuse (Figure 3). La patiente a été intubée ventilée et mise sous sédation par midazolam et fentanyl puis sous un traitement anti-epileptique à base de valproate de sodium et de clobazam. L'artériographie cérébrale réalisée dans le cadre du bilan étiologique et dans un objectif thérapeutique de l'hémorragie méningée avait suspectée une thrombophlébite cérébrale (Figure 4), d'où le complément scannographique injecté qui trouvait une thrombose des 2 tiers antérieurs du sinus longitudinal supérieur avec un infarctus frontal droit, un hématome intra-parenchymateux et une hémorragie méningée (Figure 5).

La patiente est mise sous anticoagulation curative à base d'héparine de bas poids moléculaire reliée par les anti-vitamine K avec la réalisation d'un bilan de thrombophilie et d'un bilan infectieux locorégional et systémique revenus négatifs. La patiente est trachéotomisée à J8 pour sevrage respiratoire avec une évolution favorable et une surveillance neurologique (clinique et par le doppler trans-crânien) demeurant normale au cours de son hospitalisation n'indiquant pas de geste de décompression. La récupération du déficit est complète au bout de trois semaines sans épilepsie secondaire.

## Discussion

La thrombophlébite cérébrale est une pathologie rare mais potentiellement mortelle. Elle représente 1-2% des accidents vasculaires cérébraux chez les jeunes adultes avec une incidence variant de 3 à 4 cas par million d´habitants [1]. Le tableau clinique est très varié et souvent trompeur. Les cinq signes les plus fréquents sont les céphalées (dans plus de 80% des cas), les déficits focaux, les convulsions (généralisées ou non), l'oedème papillaire et les troubles de la conscience allant de la confusion au coma profond [2].

Dans la littérature on retrouve de multiples facteurs de prédisposition à la thrombophlébite cérébrale. Il s´agit notamment de la grossesse, la prise de contraception, la déshydratation, certains médicaments, troubles de la coagulation héréditaires, la maladie systémique, le traumatisme crânien, l'infection locorégionale ou systémique, et les causes idiopathiques [3, 4]. Chez notre malade, la contraception orale était retenue comme le facteur favorisant vue la négativité des autres examens complémentaires.

Les mécanismes physiopathologiques de la TVC sont les mêmes que la thrombose veineuse profonde (troubles de l'hémostase, stase veineuse et anomalie pariétale du vaisseau) [5]. L’évolution de notre patiente suit typiquement l’évolution des différents phénomènes physiopathologiques de la TVC et de sa transformation hémorragique. La thrombose des veines cérébrales engendre localement une stase veineuse responsable d'une hypoxie tissulaire par diminution du débit sanguin cérébral qui entraîne à son tour une ischémie et un oedème cytotoxique. Les veines sont gonflées, oedématiées et les lésions d'ischémie et les lésions pétéchiales commencent à apparaître [6]. Un oedème vasogénique supplémentaire se rajoute par atteinte de la barrière hémato-encéphalique et la thrombose des sinus entraîne une augmentation de la pression veineuse, des troubles de l´absorption de liquide céphalo-rachidien, et par conséquent, une pression intracrânienne accrue [7, 8]. Le cortex et la substance blanche adjacente sont alors le siège d'une congestion, d'une hémorragie, responsable d'une souffrance cérébrale et qui s'est manifesté chez notre patiente par des crises convulsives tonico-cloniques et un état de mal épileptiques infra-clinique. Le fait que l'obstacle siège à la fin du trajet du transport du LCR et qu'aucun gradient de pression ne se développe entre les espaces sous-arachnoidiens et les ventricules explique l'absence d'hydrocéphalie dans la majorité des cas [6–8].

Le diagnostic para-clinique est établi grâce aux moyens d'imagerie. Le scanner cérébral, à lui seul, n'est pas suffisant pour affirmer le diagnostic. Il pourrait objectiver le signe direct de la thrombose en mettant en évidence le signe de Delta ou le signe du triangle dense avant l´injection du produit de contraste ou objectiver après l'injection, un réhaussement important de la paroi du sinus qui contraste avec la non injection de la lumière thrombosée (c´est le signe du delta ou du triangle vide (coupe coronale B) [9]. Les signes indirects sont surtout la conséquence de la thromboseet ils ne sont pas spécifiques de la TVC (oedème cérébral, hématome, infarctus veineux, hémorragie méningée). Ceci dit, un scanner normal n’élimine pas le diagnostic [10] et dans 40% des TVC on peut objectiver une hémorragie méningée en regard du site d'infarcissement cérébral. L'imagerie par résonance magnétique est devenue l'examen de référence pour les TVC en mettant en évidence l'occlusion vasculaire et les signes indirects en analysant son retentissement sur le tissu cérébral [11]. L’étude du liquide céphalorachidien si faite est anormale dans plus de 75% des cas et la formule la plus caractéristique est une hyperprotéinorachie généralement inférieure à un g/L, un nombre d'hématies supérieur à 20/mm3 et pléiocytose [8]. Son intérêt est surtout d’écarter une autre pathologie, de suspecter une TVC devant une hyperpression au cours de la ponction lombaire ou même thérapeutique pour la prise en charge de l'hypertension inra-crânienne. Les D-dimères, quant à leur dosage, ne permettent pas de récuser le diagnostic de TVC si négatifs ou de l'affirmer si supérieur à 500ng/ml [6]. Chez notre patiente les D-dimères demandés à postériori étaient à 1350 ng/ml. L’évolution de la TVC est imprévisible avec une mortalité estimée à 10% [12], et une incidence annuelle de récidive ou de survenue d'une thrombose veineuse cérébrale dans un autre territoire estimée à 5/100 patients [13]. Ceci-dit, certains facteurs prédictifs du décès ont été mis en évidence dans la littérature notamment un état comateux à l'admission, l’âge avancé à partir de 37, l'hémorragie méningée et les crises d’épilepsie [14]. Dans notre observation, la patiente présentait plusieurs facteurs péjoratifs mais l’évolution était favorable avec une récupération complète, cela nous pousse à songer que la prise en charge initiale rapide des thrombophlébites cérébrales et l'anticoagulation précoce même en cas d'hémorragie méningée pourraient changer le pronostic des malades.

Le traitement repose sur une prise en charge symptomatique, étiologique et surtout anti-thrombotique même en présence d'hémorragie méningée ou d'hématome parenchymateux [15]. Dans notre cas l'anticoagulation a été débutée dès la confirmation de la TVC à base de l'héparine de bas poids moléculaire en sous cutané reliée par les anti-vitamine K sans complication hémorragique notable.

## Conclusion

Malgré son faible taux d'incidence, et la diversité de ses manifestations cliniques, la thrombophlébite cérébrale reste un diagnostic à évoquer devant toute symptomatologie d'hypertension intra-crânienne, de céphalées, de troubles de consciences, d’épilépsie et aussi d'hémorragie méningée. Cette dernière ne contre-indique pas un traitement anticoagulant qui devrait être instauré en urgence dès la confirmation du diagnostic.
